# Safety, Effectiveness, and Costs of Bevacizumab-Based Therapy in Southern Spain: A Real World Experience

**DOI:** 10.1097/MD.0000000000003623

**Published:** 2016-05-13

**Authors:** Juan F. Marín-Pozo, Juan M. Duarte-Pérez, Pedro Sánchez-Rovira

**Affiliations:** From the Complejo Hospitalario de Jaén, Jaén (JFM-P, PS-R), Spain; and University of Granada (JMD-P), Granada, Spain..

## Abstract

To evaluate the safety and efficacy of bevacizumab in a broader patient population with solid tumors in the context of general clinical practice. Moreover, we quantified the economic impact and characterized the off-label use (OLU) of this agent in real-life prescribing practices.

This is an open, retrospective, observational, real world study carried out at a regional Spanish hospital attending a population of 665,000 inhabitants. All of the patients receiving bevacizumab-containing therapy between January 2006 and February 2012 at the study hospital were included: no exclusion criteria were specified. All study variables were collected from available hospital records.

The analysis comprised 240 episodes from 226 patients (male 41%; median age 57 years, 25% ≥65 years). Eighty cases (33%) of bevacizumab treatment were administered as first-line therapy. The median duration of bevacizumab treatment was 5.8 months (95% CI 5.1–6.6), without difference by age, line of treatment, or type of tumor. Typically bevacizumab-related toxicities included bleeding (25%), hypertension (5%), wound-healing complications (4%), gastrointestinal perforation (2%), and arterial thromboembolism (1%). Median progression-free survival was 7.5 months (95% CI 6.3–8.7) and median OS reached 13.1 months (95% CI 11.4–14.9). Bevacizumab increased the chemotherapy cost to 207% (from €3,115,615 to €9,552,405). Bevacizumab was prescribed off-label in 43% of episodes, amounting to €3,586,420 (56% of bevacizumab total cost).

The efficacy and safety profile of bevacizumab in routine clinical practice is consistent with results observed in prospective randomized clinical trials. OLU of this drug should be closely monitored.

## INTRODUCTION

Bevacizumab is a humanized monoclonal antibody that blocks the binding of the vascular endothelial growth factor to its receptors and results in regression of immature tumor vasculature, normalization of remaining tumor vasculature, and inhibition of further tumor angiogenesis. Because of the proposed universal antitumor activity of bevacizumab, it was widely studied in the treatment of early and metastatic tumors. Bevacizumab was first approved for treatment of advanced colorectal cancer (CRC)^1–7^ and has been approved since for advanced nonsmall cell lung cancer (NSCLC),^8–12^ renal cancer,^13–17^ and glioblastoma (GB)^18–21^ cancers by the Food and Drug Administration (FDA) in the United States. The European Medicines Agency (EMA), additionally, approved bevacizumab for treatment of other tumors such as advanced breast cancer (BC)^22–26^ (the FDA has recently revoked the recommendation for this indication), ovarian, fallopian tube, and primary peritoneal cancers;^27–29^ nevertheless, the EMA has not approved the indication of bevacizumab to treat patients with GB. The addition of bevacizumab to chemotherapy considerable increases the economic impact of the use of these agents.

The extent of inappropriate drug use is a public policy concern because of the cost and potential harms to patients from the use of toxic agents with little likelihood of clinical benefit.^30,31^ As with other more novel anticancer treatments, the off-label use (OLU) of bevacizumab (i.e., not conforming to indications listed in a drug's label as approved by Health Authorities) it is not exceptional.

The purpose of this observational study was to evaluate the safety and efficacy of bevacizumab, alone or in combination with other drugs, in a broader patient population with solid tumors in the context of general clinical practice; moreover, we quantified the economic impact and characterized the OLU of this agent in real life prescribing practices.

## PATIENTS AND METHODS

This is an open, observational, real world study carried out at a regional hospital of the Spanish National Health System attending a population of 665,000 inhabitants of southern Spain. The study was conducted in accordance with the Declaration of Helsinki and Good Clinical Practice Guidelines. The protocol was approved by the institutional review board of the hospital. Because this study was intended to reflect usual clinical practice, no compensation was provided to participating patients or physicians, and no additional assessments were required from study site or patients.

All of the patients receiving bevacizumab-containing therapy between January 2006 and February 2012 at the study hospital were included. No exclusion criteria were specified. All treatment decisions were at the physicians discretion, including dose, schedule, and duration of bevacizumab and chemotherapy, the scheduling of patient visits, and the method and frequency of clinical assessments.

All study variables were collected from available hospital records, including an oncology pharmacy application (ONCOWIN^®^) and medical history, as well as other complementary sources (pathology, laboratory, and radiology). All data were introduced in a database created ad hoc.

Information included patient age, gender, relevant medical history, cancer history (tumor type, metastatic sites, date and stage of initial diagnosis, date of advanced disease diagnosis, date of disease progression, and date of exitus), bevacizumab-based treatment (dose, schedule, line and duration of treatment, concomitant anti-cancer drugs, and best response to bevacizumab-based treatment), adverse events (AE), and number and duration of hospitalizations, either directly or indirectly related to treatment with bevacizumab. Moreover, each administration was classified as on-label if it was consistent with an EMA-approved cancer diagnosis, line of therapy, concomitant anti-cancer drugs, and dose; all other use was considered OLU.

Costs of intravenous anticancer drugs (€/mg) were obtained from the acquisition prices invoiced to the Hospital: for bevacizumab, the cost was €3.06/mg. Costs associated with hospital stay generated during bevacizumab therapy were obtained from the direct costs allocated to the oncology service by the Hospital Analytical Accountability System.^32^

All AEs were graded using the National Cancer Institute-Common Terminology Criteria for Adverse Events version 4.0 and coded according to the Medical Dictionary for Regulatory Activities.

Effectiveness measures included the progression free survival (PFS) duration (from the start of the initial bevacizumab-containing therapy to the first recorded occurrence of physician-assessed disease progression or death), overall survival (OS) duration (from the start of the bevacizumab-containing therapy to death or censoring), and response rate (using the RECIST criteria). Patients without an event who remained in follow up were censored on March 2014.

### Statistical Analysis

The mean, standard deviation, median, range, and counts and percentages (categorical data) were calculated for demographic and cancer characteristics. The overall AE incidence was summarized in terms of patient counts and 95% confidence intervals (CIs); relationship between hospital admissions and the occurrence of AEs was also calculated. The response rates were calculated with their CIs. Progression free survival and OS were expressed as a median survival with 95% CIs.

Frequencies for the adequacy to the bevacizumab summary of product characteristics and by reason for inadequacy (OLU) were determined for each administration. Average incremental costs because of adding bevacizumab to standard chemotherapy were calculated (global and by pathology).

Chi-square tests were used to compare categorical variables between treatment groups. For continuous variables, we used Student *t* tests. The Kaplan–Meier method was used to estimate survival curves, and the log-rank test was used to compare the curves. Cox proportional hazards modeling was used to calculate hazard ratios and 95% confidence intervals (CI). Statistical analyses were performed using SAS version 9.2.

## RESULTS

### Population Characteristics

Registers from 240 episodes of treatment with bevacizumab were recorded, corresponding to 226 patients: 12 patients received 2 bevacizumab-based treatment lines, and 1 patient received 3 lines.

Patient and tumor characteristics are summarized in Table 1. A quarter of patients were ≥65 years old. Most of patients had ≥ 2 metastatic sites (127 patients, 53%). Median annual incidence of new bevacizumab-based treatments was 34 (range 9–64), with a progressive increase of cases from 2006 (9 cases) to 2012 (64 cases).

**TABLE 1:**
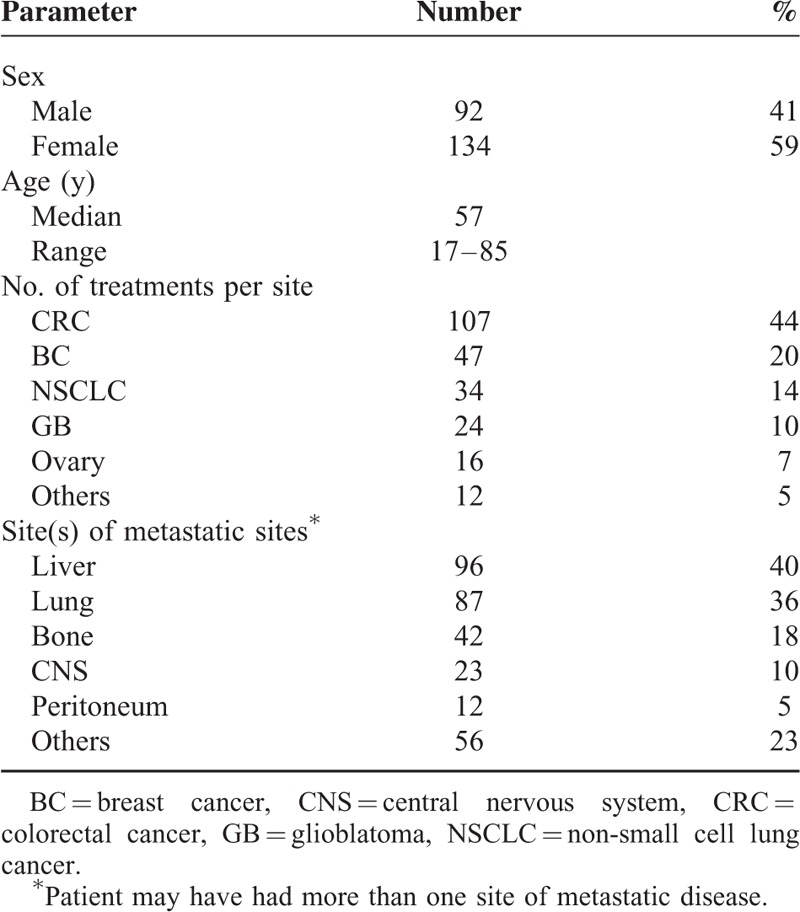
Population and On-Study Treatment Characteristics

Eighty cases of bevacizumab treatment were administered as first-line therapy (35 CCR, 16 BC, and 29 NSCL; 33% of cases) and 160 cases (67%) as second or later line. Patients received a mean of 13 (range, 1–80) doses of bevacizumab, with a mean dose of 631 mg per patient (range 232–1506 mg).

Chemotherapy regimens combined with bevacizumab varied depended on the tumor: in CRC, most of combinations included irinotecan (48 cases), oxaliplatin (30 cases), and fluoropirimidinas (18 cases), whereas in 11 cases, bevacizumab was administered as monotherapy; patients with BC received bevacizumab in combination with a taxane in 37 cases (29 paclitaxel and 8 docetaxel), with others drugs in 7 cases, and as monotherapy in 3 cases; in NSCLC the more frequent regimen was bevacizumab+carboplatin+paclitaxel (21 cases), following by bevacizumab+cisplatin+gemcitabine (9 cases), bevacizumab+permetrexed (2 cases), and bevacizumab in monotherapy (2 cases); patients with ovarian cancer received bevacizumab+topotecan in 6 cases, bevacizumab+liposomal doxorubicine in 4 cases, bevacizumab+oxaliplatin in 1 case, and bevacizumab as monotherapy in 5 cases; in GB 21 cases were treated with bevacizumab+irinotecan and 3 cases received bevacizumab as monotherapy.

The median duration of bevacizumab treatment was 5.8 months (95% CI 5.1–6.6). There was no statistically significant difference in the duration of treatment based on age, line of treatment, and type of tumor.

### Response to Treatment

In 35 cases no response was documented, so 205 cases were considered evaluable. The best overall response rate (ORR) was 48% (99 patients, 95%CI 42–55), including 6% of complete response (12 patients). The clinical benefit rate (ORR+disease stabilization rate) was 72% (147 patients, 95%CI 65–77). Response rates by tumor and line of treatment are shown in Table 2. Median PFS for the entire study population was 7.5 months (95% CI 6.3–8.7) and was similar in patients aged ≤65 years (7.5 months, 95% CI 5.6–9.4) than in patients aged >65 years (6.8 months, 95% CI 4.8–8.9) (*P* = 0.28). The median OS time was 13.1 months (95% CI 11.5–14.8), 13.6 months (95% CI 12.1–15.1) in ≤65 years and 11.4 (95% CI 7.0–15.8) in >65 years (*P* = 0.72). Median PFS and OS by type of tumor are shown in Table 3.

**TABLE 2:**
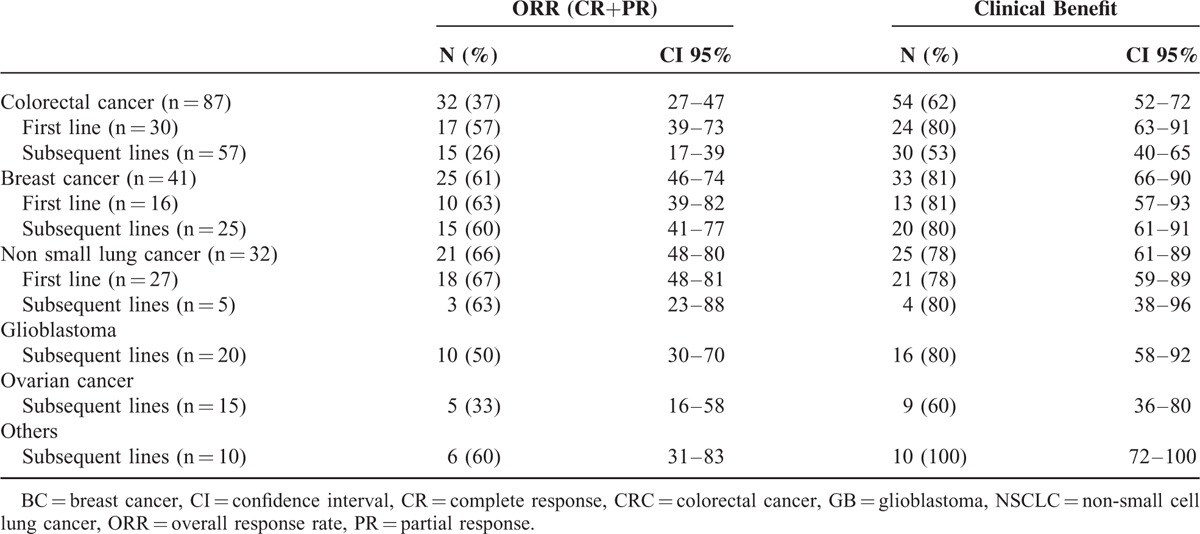
Response Rates by Type of Tumor and Line of Treatment in 205 Evaluable Patients

**TABLE 3:**
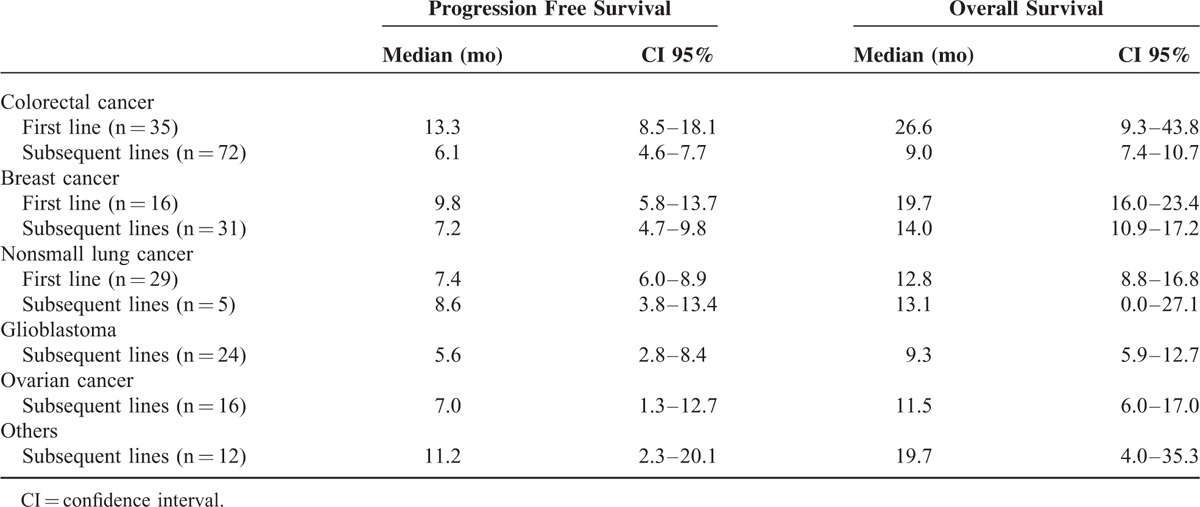
Progression Free Survival and Overall Survival by Type of Tumor and Line of Treatment

### Safety

**FIGURE 1:**
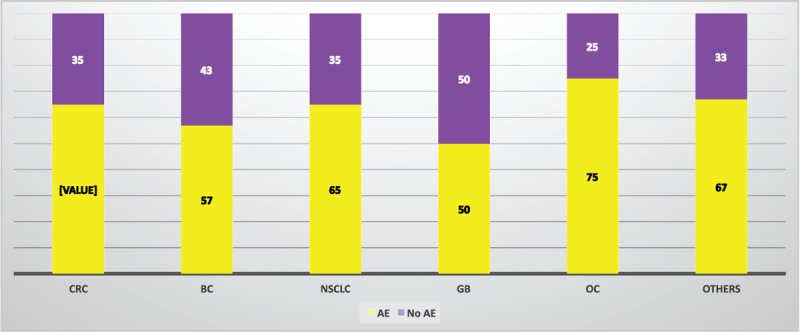
Adverse events per cancer type. AE = adverse event, BC = breast cancer, CRC = colorectal cancer, GB = glioblastoma, NSCLC = nonsmall cell lung cancer, OC = ovarian cancer.

Any treatment-related toxicity was reported in 150 patients, ranging from 50% of patients with GB to 75% of ovarian cancer subjects (Figure 1). The most frequent treatment-related adverse events (occurring at a frequency >5% of patients) are shown in Table 4. Between the typically bevacizumab-related toxicities, in addition to bleeding (most of them were mild nose or gum bleeding), hypertension was reported in 12 patients (5%), wound-healing complications in 9 patients (4%), gastrointestinal perforation in 5 patients (2%), and arterial thromboembolism in 3 patients (1%). Twenty-nine patients (12.1%) discontinued treatment with bevacizumab because of AEs (Table 5). A CRC patient died within the first 24 hours after receiving the 8th doses of bevacizumab: cause of death could not be determined. Forty-six patients experiencing any AE required hospitalization: a statistically significant association between AE and hospitalization was found (chi-square = 9.25, *P* = 0.002). Patients without AE were hospitalized for a total of 62 days, with a cost of €17,276; patients with any AE counted 646 days of hospitalization, with a cost of €180,002.

**TABLE 4:**
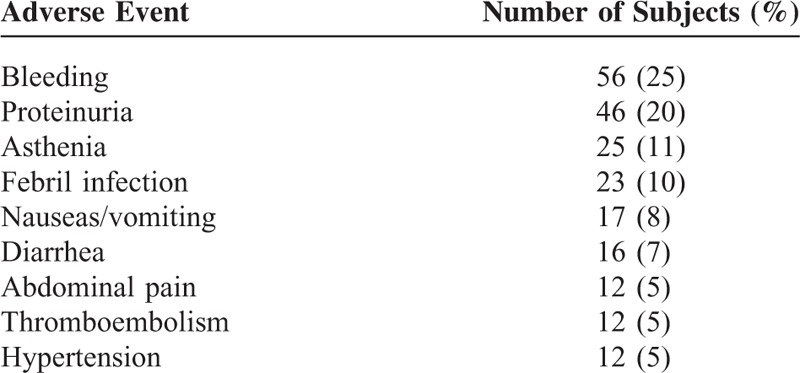
Adverse Events Related to Bevacizumab (≥5%) Per Patient

**TABLE 5:**
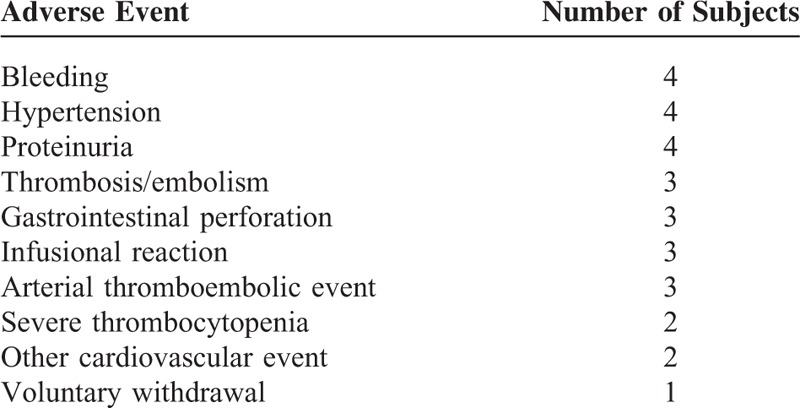
Adverse Events Leading to Bevacizumab Discontinuation

### Treatment Costs and Off-Label Use

**FIGURE 2:**
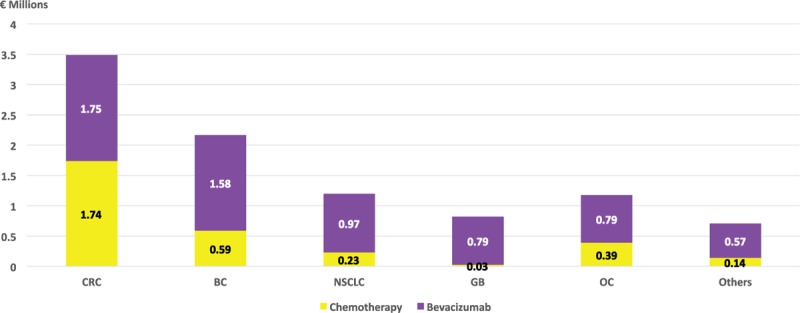
Total costs per cancer type (€ millions). BC = breast cancer, CRC = colorectal cancer, GB = glioblastoma, NSCLC = nonsmall cell lung cancer, OC = ovarian cancer.

Total cost of bevacizumab-related treatment was €9,552,405, with a median of €39,802 per treatment. The cost of acquisition of bevacizumab was €6,436,790 (67% of total treatment cost), representing a 207% increment over the chemotherapy cost. Incremental costs per treatment because of bevacizumab ranging from €16,366 in CRC to €49,182 in ovarian cancer, with an average increase of €26,820 per treatment (Figure 2).

One hundred three episodes of treatment with bevacizumab were considered OLU (43%, 95% CI 37–49), mainly because of lack of approval for the specific disease (35 episodes, 24 of them gliomas), concomitant treatment (35 episodes: in 21 of them it was not combined with fluoropyrimidines in CRC and in 10 cases was used in monotherapy to treat breast or ovarian cancer), or line of treatment (31 episodes, 26 of them in second-line of BC and 5 in second-line of NSCLC). Total cost of bevacizumab-related OLU treatment was €4,858,334 (51% of 240 treatments cost); incremental costs because of bevacizumab OLU treatment amounted to €3,586,420 (56% of bevacizumab total cost).

## DISCUSSION

Translating results from randomized clinical trials of new drugs into general clinical practice can be challenging, because those results are based on selected patients who may not fully represent the general patient population treated in the community.

This observational cohort study collected outcomes data from a general population of oncologic patients, thereby expanding information on the safety and effectiveness of bevacizumab. Reflecting current chemotherapy usage patterns in Europe, bevacizumab was most often paired with FOLFIRI and FOLFOX in metastatic CRC, and with carboplatin–paclitaxel in metastatic NSCLC. We got an overall clinical benefit rate of 82%, a median PFS time of 7.5 months, and a median OS of 13.1 months, with no significant differences between younger (<65 years) and older (≤65 years) people, and an expected safety profile. The addition of bevacizumab to chemotherapy incremented the cost of treatment 207% over the chemotherapy cost, and 43% of 240 bevacizumab-based treatments were OLU (56% of bevacizumab total cost).

The median PFS was similar to that reported in clinical trials for most of solid tumors.^33^ Noteworthy, the PFS reached in first line treatment of patients with metastatic CRC is among the higher reported in clinical trials. In front line of metastatic BC median PFS found in our study was slightly lower than that of the E2100 study,^24^ but similar to that of the pooled analysis of three randomized phase III trials in HER2-negative patients^34^: this may be because of the existence of a large group of patients with poor prognosis factors such as triple negative subtype^35^ or inflammatory BC.^36^

Overall, we found no difference in the effectiveness of bevacizumab between the two age groups (<65 or >65 years). Probably, this fact reflects the good tolerance of bevacizumab. Nevertheless, in patients with NSCLC receiving bevacizumab as first-line treatment, PFS [≤65 years (8.6 months, 95% CI 0.0–22.7), >65 years (6.5 months, 95% CI 5.4–7.7) (*P* = 0.05)] and OS [≤65 years (18.0 months, 95% CI 13.2–22.8), >65 years (10.7 months, 95% CI 6.9–14.5) (*P* = 0.36)] tended to be more prolonged in younger people. These data are in line with those reported in clinical trials and observational studies,^37,38^ where the addition of bevacizumab to paclitaxel–carboplatin was associated with a higher degree of toxicity, but no obvious improvement in survival compared with paclitaxel–carboplatin in elderly NSCLC patients.

Despite having several unfavorable prognostic factors at baseline, such as the presence multiple metastatic sites and the number of prior lines of therapy, this cohort exhibited no substantial differences in the reported proportions of patients experiencing the known bevacizumab-related AEs.^2,12,24,28^ The incidence of side effects was higher in CRC, NSCLC, and ovarian cancer, which would agree with the existence of a disease specific security pattern, as has been postulated by others.^33^ In the meta-analysis of Ranpura et al, the addition of bevacizumab to chemotherapy or biological therapy was associated with increased treatment-related mortality^39^: nevertheless, in our study, only one death was related to treatment, and probably was because of progression of disease.

The addition of bevacizumab to chemotherapy represents an important increase in the cost of treatment and, as indicated in other studies, this drug (similarly to other monoclonal antibodies such as cetuximab and panitumumab) does not seem to be cost effective.^40^ Metastatic CRC is the most frequent tumor where bevacizumab is used and where the lowest incremental cost for this drug was got in our study (€1303 per dose and day). Following the economic model developed by Dranitsaris et al for Spanish hospitals,^41^ a price of €244 per daily dose and month survival benefit would be considered cost effective for metastatic CRC in Spain. In this study, an increase of 5.1 months survival (26.6 months) was reached over the best survival data of GERCOR study (21.5 months),^42^ so bevacizumab would be cost effective if had a cost <€1245 per daily dose. Therefore, although close to the threshold of efficiency, bevacizumab did not reach it in that disease.

We found a high proportion of bevacizumab treatments being OLU (43%). Joerger et al,^43^ using similar criteria to those used in our study, found 30% of OLU of bevacizumab in eastern Switzerland, mainly because of its use in treating advanced ovarian cancer beyond the second-line setting and advanced breast cancer beyond the first-line setting. A study conducted in the United States^44^ showed an OLU of bevacizumab of 52%, in spite of the OLU criteria were less strict than in our study. Furthermore, they estimate that sales for OLU of bevacizumab accounted for 62.6% of a total of 3100 million dollars in sales of bevacizumab in that country. As in our study, the cost of OLU of bevacizumab was higher than that used on-label, probably because of major utilization of the 5 mg/kg/wk bevacizumab doses when this drug was used off-label.

This study has several limitations, some of them linked to the observational design. Investigators were instructed to recruit all eligible patients for participation in the study. Because investigators were not required to maintain a list of patients who were eligible but not enrolled, the potential for bias in patient selection could not be evaluated. As an observational study, the frequency of response assessments was not defined in the protocol but determined by individual investigators, so less frequent response assessment than the clinical trial standard of every 8 weeks could bias in favor of a longer median PFS time. On the contrary, the dissimilarity in the chemotherapy regimens, dosing, and schedules make difficult to do a global analysis. The heterogeneous length of treatment and follow up contribute to the asymmetry as well. Also, the small number of patients in some lines of treatment and/or kind of tumor difficult the extrapolation of the outcomes. As postulated by others,^33^ the biological rationale to combine all studies in order to assess bevacizumab universal effect might offset these limitations.

## CONCLUSION

Our results suggest that, in line of randomized clinical studies, bevacizumab in the clinical practice adds activity to several chemotherapy regimens in a wide range of tumors, even in an unselective cohort of patients.

Bevacizumab, a high-cost monoclonal antibody, is used off-label frequently. Off-label use (OLU) of this drug should be closely monitored, taking in mind the costs and potential toxicity that can be associated with OLU of this drug.
